# Zooplankton diversity monitoring strategy for the urban coastal region using metabarcoding analysis

**DOI:** 10.1038/s41598-021-03656-3

**Published:** 2021-12-21

**Authors:** Chi-une Song, Hyeongwoo Choi, Min-Seung Jeon, Eun-Jeong Kim, Hyeon Gyeong Jeong, Sung Kim, Choong-gon Kim, Hyenjung Hwang, Dayu Wiyati Purnaningtyas, Seok Lee, Seong-il Eyun, Youn-Ho Lee

**Affiliations:** 1 Department of Life Science, Chung-Ang University, 84 Heukseok-ro, Dongjak-gu, Seoul, 06974 Korea; 2 Department of Taxonomy and Systematics, National Marine Biodiversity Institute of Korea, Seocheon-gun, Chungchungnam-do 33662 Korea; 3 Korea Institute of Ocean Science and Technology, 385 Haeyang-ro, Yeongdo-gu, Busan, 49111 Korea; 4 KIOST School, University of Science and Technology, Busan, 49111 Korea

**Keywords:** Environmental sciences, Ocean sciences

## Abstract

Marine ecosystems in urban coastal areas are exposed to many risks due to human activity. Thus, long-term and continuous monitoring of zooplankton diversity is necessary. High-throughput DNA metabarcoding has gained recognition as an efficient and highly sensitive approach to accurately describing the species diversity of marine zooplankton assemblages. In this study, we collected 30 zooplankton samples at about 2-week intervals for 1 year. Zooplankton diversity showing a typical four season pattern. Of the “total” and “common” zooplankton, we assigned 267 and 64 taxa. The cluster structure and seasonal diversity pattern were rough when only the “common” zooplankton was used. Our study examined how to maximize the benefits of metabarcoding for monitoring zooplankton diversity in urban coastal areas. The results suggest that to take full advantage of metabarcoding when monitoring a zooplankton community, it is necessary to carefully investigate potential ecosystem threats (non-indigenous species) through sufficient curation rather than disregarding low-abundance operational taxonomic units.

## Introduction

Zooplankton play a key role in marine biodiversity and thus have critical impacts on marine ecosystem processes^1–4^. These animals are integral to the functioning of aquatic food webs because they constitute the major link for energy transfer between phytoplankton, the primary producers, to higher species and further to predators, such as commercially important fish larvae^5–7^. Hence, information on zooplankton communities and diversity is an important aspect of understanding marine ecosystems. Most of the fluctuations in zooplankton communities are caused by environmental factor changes and the relatively short life-cycle of zooplankton (from a few months to 1 year). Therefore, sampling at 2-week to 1-month intervals can be sufficient to track the changes in the marine environment’s seasonal and interannual conditions both directly and indirectly^8^.

Metabarcoding has revolutionized biomonitoring in marine and freshwater ecosystems^4,9^. Not only has metabarcoding allowed researchers to examine the relationship between environmental changes and aquatic communities in benthic environments^10,11^, but it is also a highly effective approach for large-scale biodiversity assessment, large-scale community structure, and diversity analysis of zooplankton in the oceanic zones of the Pacific Ocean and the Arctic Ocean^6^. Research using metabarcoding can help overcome some of the weaknesses of traditional analysis. Metabarcoding analysis has high-throughput sequencing sensitivity and can discriminate cryptic species and rare species with low abundances such as early invaders that would be missed in traditional classification^12,13^. Furthermore, it can be useful when it is difficult to identify the morphological classification key such as physically damaged samples or the larval stage of invertebrates, which can decrease the classification’s resolution. In addition, metabarcoding is more practical and cost-effective than the traditional method which requires many experts’ labor and time to study the wide diversity of marine zooplankton^3,4,9,14–16^. Due to these advantages, it is essential to study zooplankton diversity using metabarcoding on a global to local scale.

Urbanized coastal areas may have various harmful influences on the original ecosystems due to the population increase and the artificial development of ports and reclamation areas. Therefore, continuous monitoring of ecosystem and biodiversity changes is necessary. In the urbanized and industrialized coastal inner bays and ports, environmental changes frequently occur because of artificial factors such as pollutants from cities or ships^17^. Alien species in the ballast water of large vessels that traverse the ocean may also disturb ecosystem^9,18,19^. Continuous monitoring of the zooplankton community and diversity in the region will provide useful data in responding to human activities and ecosystem changes and crises. Thus, we chose the biggest port, Busan in Korea. The northeastern part of Yeongdo-gu, Busan (South Korea), is actively urbanized. It is the entrance to Busan Port, one of the world’s largest ports and is visited by many ships. Busan port development, reclamation, and installation of water breakers have decreased the length of the natural coastal line and velocity of seawater flow in this area^20^. Potential and persistent environmental pollution from the increase in human activities in this area can also cause marine ecosystem instability^21^.

**Figure 1:**
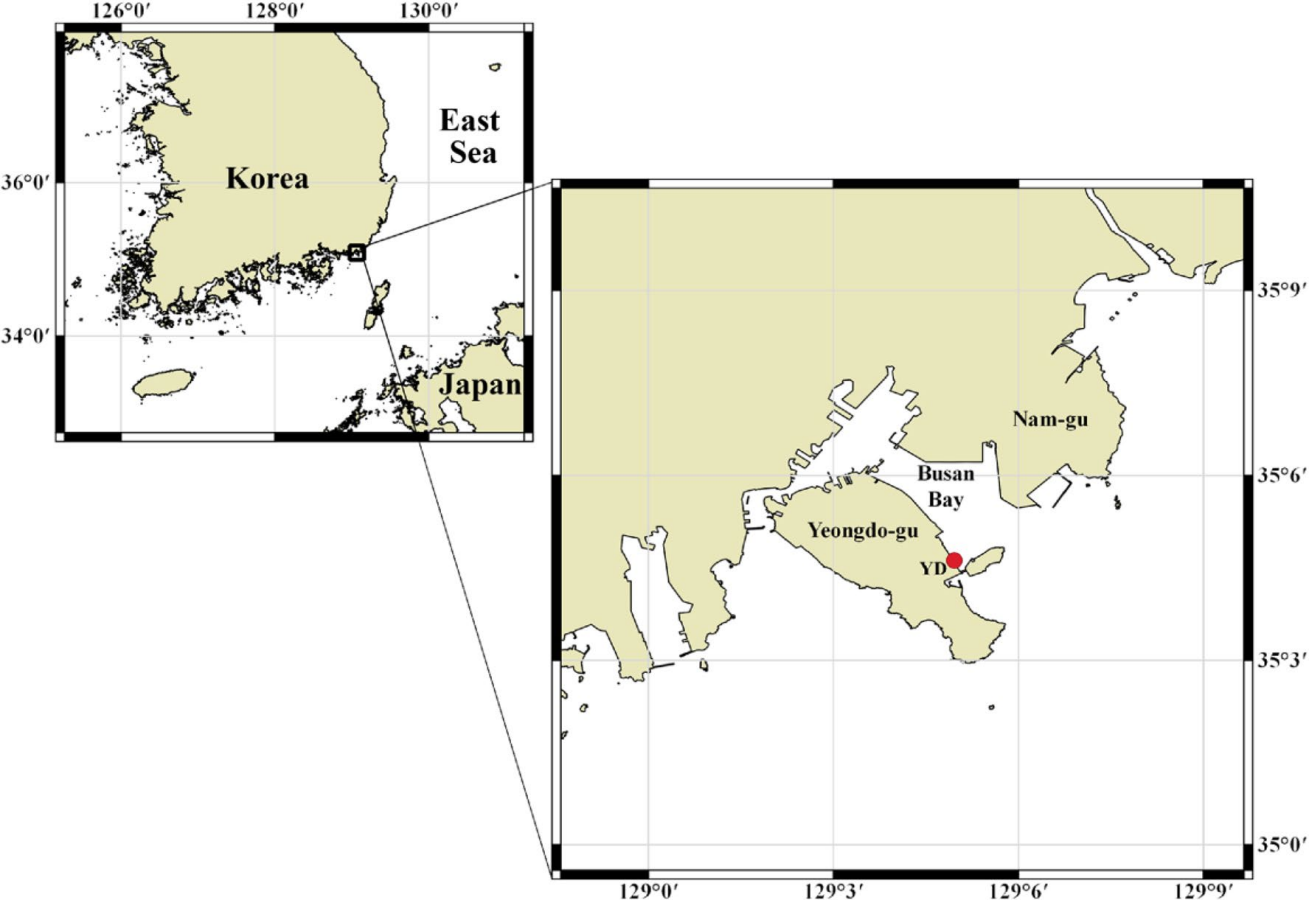
Location of the sampling site (red dot) in Busan Bay, southern coast of Korea. The maps were created using QGIS (v.3.16; www.qgis.org). The base map is from OpenStreetMap and OpenStreetMap Foundation under the Open Database License (https://www.openstreetmap.org/copyright).

In the current study, zooplankton samples collected at 2-week intervals from February 2019 to April 2020 from a sampling station in Yeongdo-gu, Busan, were analyzed by metagenomics to reveal the pattern of changes in zooplankton diversity and community structure over time (Fig. 1; Table 1). Using “common” and “total” zooplankton data, we investigate the process most suitable for low-abundance operational taxonomic units (OTUs) when performing comprehensive and long-term coastal ecosystem monitoring. In addition, we compare the species identified in this study with previously reported species and choose candidates for potential non-indigenous species (NIS) that could cause a disturbance in the marine ecosystem. Furthermore, we discuss the limitations of zooplankton diversity studies that use molecular methods and how to overcome these problems and improve accuracy in the future. The results from this study will play an important role in studying zooplankton diversity and long-term variability in the port area.

**Table 1:** Sample information and summary of reads.

Sample	Sampling date	Total number of NGS raw sequences	Number of sequences after filtering	Number of sequences assigned OTUs	NCBI accession
YD2	Feb-13-2019	348,222	310,695	287,126	SAMN19778297
YD4	Feb-27-2019	395,508	364,154	337,084	SAMN19778298
YD6	Mar-13-2019	338,421	310,003	290,928	SAMN19778299
YD8	Mar-27-2019	357,906	331,215	308,353	SAMN19778300
YD10	Apr-11-2019	399,782	371,355	326,538	SAMN19778301
YD12	Apr-24-2019	399,022	368,531	338,928	SAMN19778302
YD14	May-08-2019	367,517	339,868	323,339	SAMN19778303
YD15	May-22-2019	367,072	340,784	305,939	SAMN19778304
YD18	Jun-05-2019	310,710	288,350	270,518	SAMN19778305
YD20	Jun-19-2019	350,252	325,138	296,833	SAMN19778306
YD22	Jul-03-2019	361,334	336,418	305,988	SAMN19778307
YD26	Jul-31-2019	425,773	393,379	361,751	SAMN19778308
YD28	Aug-14-2019	417,705	395,316	342,677	SAMN19778309
YD30	Aug-28-2019	396,455	370,929	339,889	SAMN19778310
YD32	Sep-10-2019	374,029	355,505	321,004	SAMN19778311
YD34	Sep-24-2019	363,488	342,630	308,631	SAMN19778312
YD36	Oct-10-2019	357,907	339,227	294,752	SAMN19778313
YD38	Oct-23-2019	303,003	281,268	246,404	SAMN19778314
YD40	Nov-06-2019	166,190	146,654	123,777	SAMN19778315
YD42	Nov-20-2019	294,588	272,311	248,292	SAMN19778316
YD44	Dec-05-2019	271,965	252,448	237,477	SAMN19778317
YD46	Dec-18-2019	193,673	181,096	165,954	SAMN19778318
YD48	Jan-02-2020	120,931	110,537	99,281	SAMN19778319
YD50	Jan-15-2020	203,514	190,278	172,328	SAMN19778320
YD52	Jan-29-2020	174,310	162,272	145,458	SAMN19778321
YD54	Feb-13-2020	264,813	242,973	186,811	SAMN19778322
YD56	Feb-26-2020	97,579	88,304	81,811	SAMN19778323
YD58	Mar-18-2020	270,833	250,178	230,453	SAMN19778324
YD60	Apr-01-2020	271,377	245,750	228,305	SAMN19778325
YD62	Apr-16-2020	200,092	182,873	155,197	SAMN19778326
Total		9,163,971	8,490,439	7,681,826	

## Results

### Environmental conditions and summary of DNA data and taxonomic assessment

Water temperature and salinity were measured at the same station and on the same dates as most zooplankton samplings from February 2019 to March 2020 at 2-week intervals. The average water temperature was 16.6 ℃, ranging from 11.4 to 27.6 ℃ (see Fig. S2). It gradually increased from February 2019, peaked on August 14, 2019, and then decreased. The average water temperatures in February and March of 2020 were slightly lower than those in 2019. During the same sampling period, the salinity (practical salinity unit, psu) ranged from 30.2 to 34.5 psu, with an average of 33.0 psu. Contrary to water temperature, salinity generally decreased and increased again from February 2019 to September 2019 (Table S1; Fig. S2). These seasonal changes in water temperature and salinity are consistent with previous studies in the Busan Bay and the Southern coast of Korea^22–25^.

A total of 9,163,971 amplicons were sequenced from the 30 samples and 8,490,439 reads (92.7%) remained after the stringent quality filtering of chimeras (Table 1). The number of OTUs (at the 98% similarity level) was 4,204 for all samples, varying between 166 and 601 OTUs for each sample (Table S1). Sequence reads were normalized to the minimum reads per sample (81,811 reads, YD56) for a sample-to-sample analysis due to different samples showing different numbers of sequence reads (Fig. S3a). After rarefying, the number of OTUs was 3,486 for all samples, varying between 147 and 473 OTUs for each sample (Table S1). After BLAST search to the NCBI nt database, 426 OTUs (932,472 reads) were retained. Approximately 63% (267 OTUs and 771,849 reads) were classified as zooplankton taxa from the taxonomic assessment (Tables S1 and S2; Fig. S4). The proportion of reads for the zooplankton communities in each sample was summarized in Fig. S4 and Table S2. Of 267 “total” zooplankton OTUs, 72 taxa were identified as copepods, representing 38.6% of the total reads. The BLAST scores and modified zooplankton species name were listed in Table S3.

Filtering for common OTUs contributing > 0.5% of sequence reads in at least one sample resulted in 239 OTUs, representing 95% of the total reads. Finally, 64 “common” zooplankton taxa were classified from the taxonomic assessment (Table S1). Rarefaction was performed after taxonomic classification because the rarefaction curve reached a plateau due to the decrease in rare OTUs (Fig. S3b).

### Seasonal trends of α-diversity and taxonomic composition of zooplankton based on metabarcoding data

**Figure 2:**
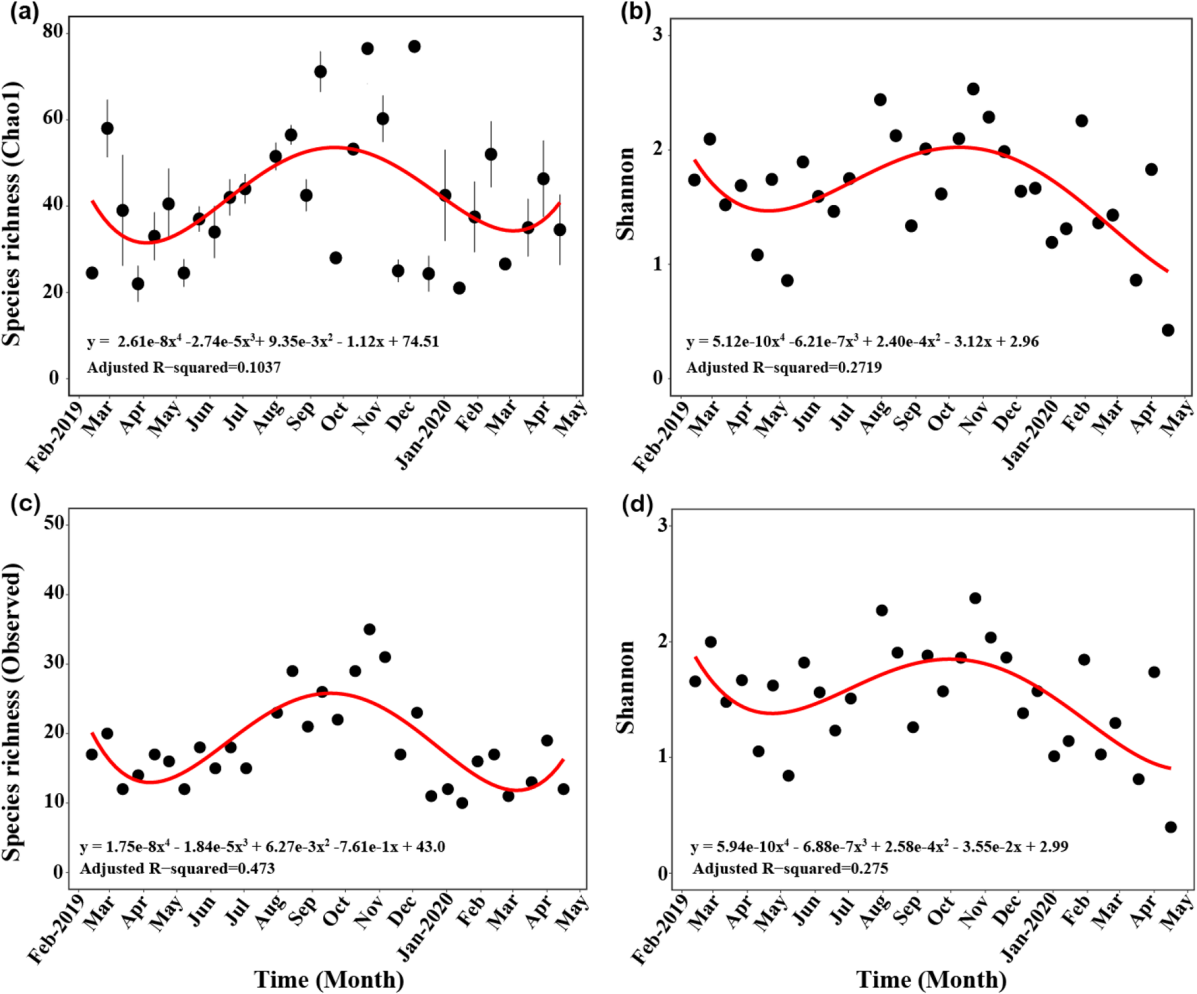
Temporal distribution of the α-diversity (species richness and Shannon index). (**a**) Chao1 index for “total” zooplankton species. (**b**) Shannon diversity index for “total” zooplankton. (**c**) Observed species richness for “common” zooplankton. (**d**) Shannon diversity index for “common” zooplankton. Standard errors and the regression lines are indicated by the vertical and red lines. Figures were produced using R (v4.0.3, https://www.R-project.org).

The average Chao1 index of “total” zooplankton was 42, ranging from 21.00 ± 0.16 (YD50) to 77.00 ± 30.34 (YD44). The overall trends of the Chao1 index was low in April and high in September (Fig. 2a; Table S4). The average Shannon diversity index of “total” zooplankton was 1.66, which varied from 0.43 (YD62) to 2.53 (YD38). Similar to the Chao1 index, the overall trends of the Shannon diversity was low in April and high in September to October, showing a seasonal pattern. However, diversity declined slightly in early 2020 compared with a similar period in 2019 (Fig. 2b; Table S4). The observed taxa and Shannon diversity index of “common” zooplankton had similar distribution patterns to those of “total” zooplankton throughout the sampling period. However, the R^2^-value of the polynomial regression analysis for the observed taxa of “common” zooplankton was higher than that for the Chao1 index of “total” zooplankton (Fig. 2c, d; Table S4).

With the environmental data measured on the zooplankton sampling day (accepting ± one date gap), the correlation between the α-diversity index (Chao1 and Shannon) and environmental factors was confirmed. The α-diversity indices were positively correlated with water temperature and negatively correlated with salinity (Fig. S5). The α-diversity showed a slightly higher correlation with water temperature than salinity because salinity was more conserved throughout the year (Fig. S5). Seasonal pattern was analyzed by dividing into three main mesozooplankton groups^4,24^; copepods (average of 47%), meroplankton (43%), and non-copepod holoplankton (10%) (Fig. S6). The three groups showed highly seasonal dynamic patterns; the dominant group was copepods in January (average of 94.5%) and Meroplankton in September (88.89%).

**Figure 3:**
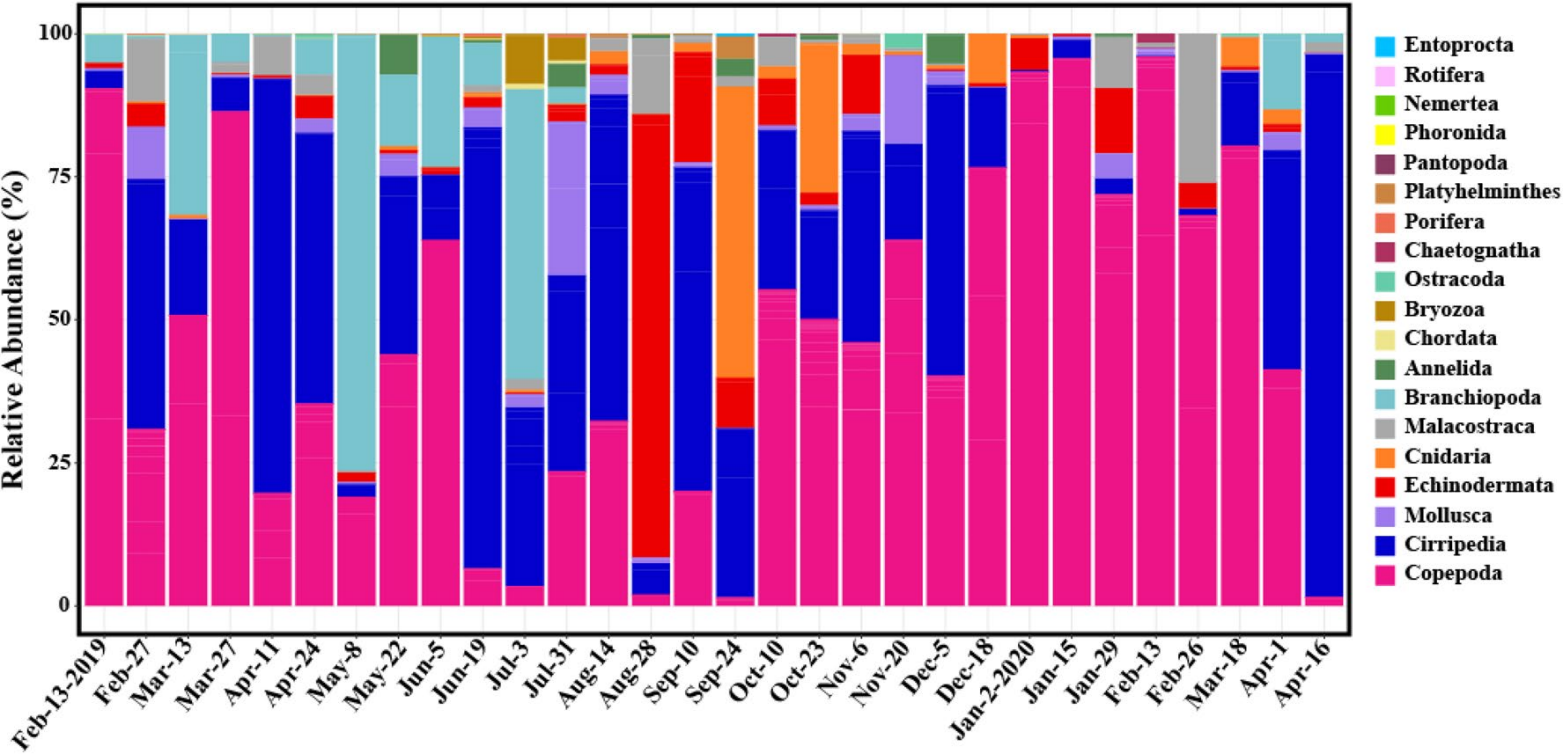
Taxonomic composition of zooplankton for 30 samples with 19 taxonomic groups. The bar heights are indicated proportion (percentage of reads) of each taxonomic group. Figures were produced using R (v4.0.3, https://www.R-project.org).

To confirm the overall species composition, the total number of species was divided into 19 taxonomic groups at the phylum or class level (subclass or infraclass). The relative abundances in each sample were shown as a bar plot (Fig. 3; Table S5). Copepoda (average of 47%) and Cirripedia (28%) appeared in all 30 samples. Additionally, the frequency of reads was relatively high in Branchiopoda (7.9%, *n* = 23 where *n* is the number of samples appeared), Echinodermata (5.7%, *n* = 29), Cnidaria (3.6%, *n* = 27), Malacostraca (3.0%, *n* = 26), and Mollusca (2.8%, *n* = 29). The remaining 12 taxonomic groups (Annelida, Bryozoa, Chaetognatha, Chordata, Entoprocta, Nemertea, Ostracoda, Pantopoda, Phoronida, Platyhelminthes, Porifera, and Rotifera) were relatively rare taxonomic groups (average of relative abundance < 1%). When the same analysis was performed with only “common” zooplankton species, the results were similar to the above but only 11 taxonomic groups remained (Fig. S7).

**Figure 4:**
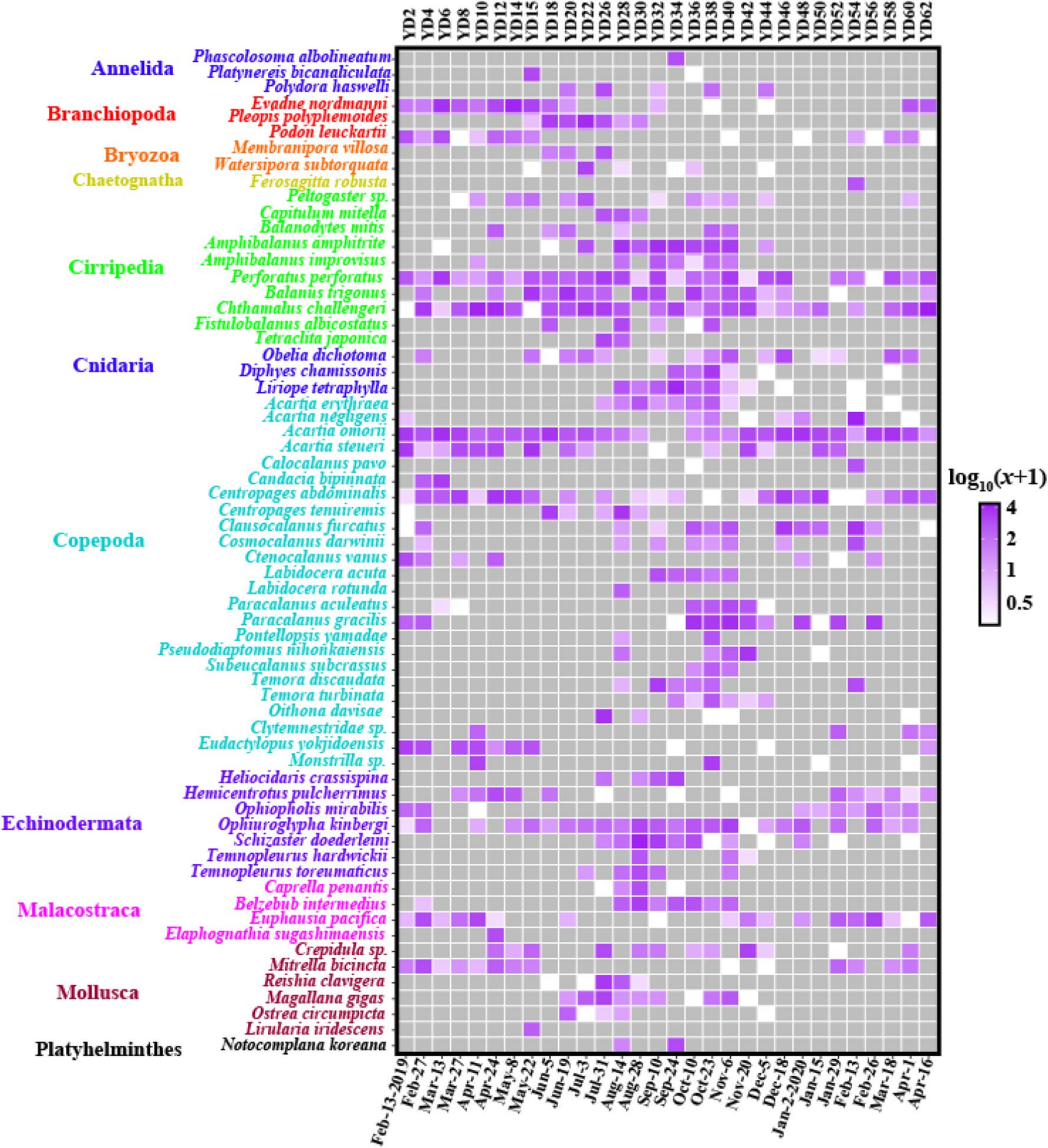
Heatmap of 64 “common” zooplankton taxa. Each read count is transformed log_10_(abundance + 1). The colors indicate relative abundance from high (purple) to low (white), and gray is 0. Figures were produced using R (v4.0.3, https://www.R-project.org).

The temporal distribution patterns of “common” zooplankton were indicated in a heatmap (Fig. 4; Table S6). Copepod species remained the most dominant with 24 taxa from 64 zooplankton taxa (Fig. S8) followed by Cirripedia with 10 species. *Chthamalus challengeri* (Cirripedia) (mean of abundance [MA]: 2.2; the number of samples in which this species existed [NS]: 27) appeared dominantly throughout the sampling periods and *Perforatus perforatus* (MA: 2.0, NS: 28) and *Balanus trigonus* (MA: 1.3, NS: 17) followed. Seven species of Echinodermata were identified. *Ophiuroglypha kinbergi* was found in most samples (MA: 1.5, NS: 24) and *Schizaster doederleini* (MA: 0.7, NS: 12) existed only in 12 samples between July and October with high average abundance. Six molluscan taxa were identified of which *Mitrella bicincta* (MA: 0.7, NS: 14) was the most dominant in winter. Four Mollusca taxa (*Magallana gigas*, *Reishia clavigera*, *Ostrea circumpicta*, and *Crepidula* sp.) were more prevalent in summer than in winter. The Mollusca species *Lirularia iridescens* was found only once on May 22. There are 4 taxa in Malacostraca. *Euphausia pacifica* (MA: 0.9, NS: 18) was the most prevalent and appeared mainly in winter, while *Belzebub intermedius* was found mainly between August and October (MA: 0.5, NS: 8). Three species of Branchiopoda were identified. *Evadne nordmanni* (MA: 1.1, NS: 15) and *Podon leuckartii* (MA: 0.6, NS: 15) appeared mainly between January and May, while *Pleopis polyphemoides* (MA: 0.5, NS: 7) appeared relatively briefly, emerging between May and August. The remaining taxa also showed different distribution patterns along the sampling period. For data on all 64 “common” zooplankton species (Table S6).

**Figure 5:**
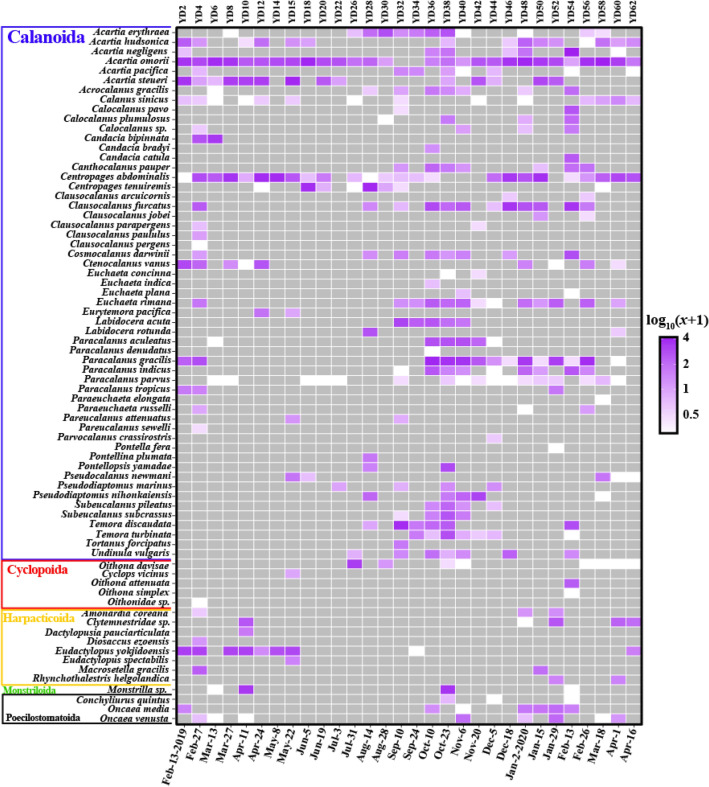
Heatmap of subclass Copepoda. Each read count is transformed log_10_(abundance + 1). The colors indicate relative abundance from high (purple) to low (white), and gray is 0. Figures were produced using R (v4.0.3, https://www.R-project.org).

Copepods were the most prevalent and abundant group in our samples. They consisted of five orders (Calanoida, Cyclopoida, Harpacticoida, Monstrilloida, and Poecilostomatoida) and their temporal distributions were visualized in a heatmap (Fig. 5). With 72 taxa (68 species, 2 genera, 2 families), Calanoida (54 species) showed the most common order followed by Harpacticoida (7 species), Cyclopoida (4 species), Poecilostomatoida (3 species), and Monstrilloida (1 species). The most prevailing species (that which appeared in most samples) was *Acartia omorii* (MA: 2.7, NS: 28) followed by *Centropages abdominalis* (MA: 1.6, NS: 25). Each of the others showed various temporal distribution patterns (Fig. 5; Table S7).

### Seasonal differences in zooplankton communities

**Figure 6:**
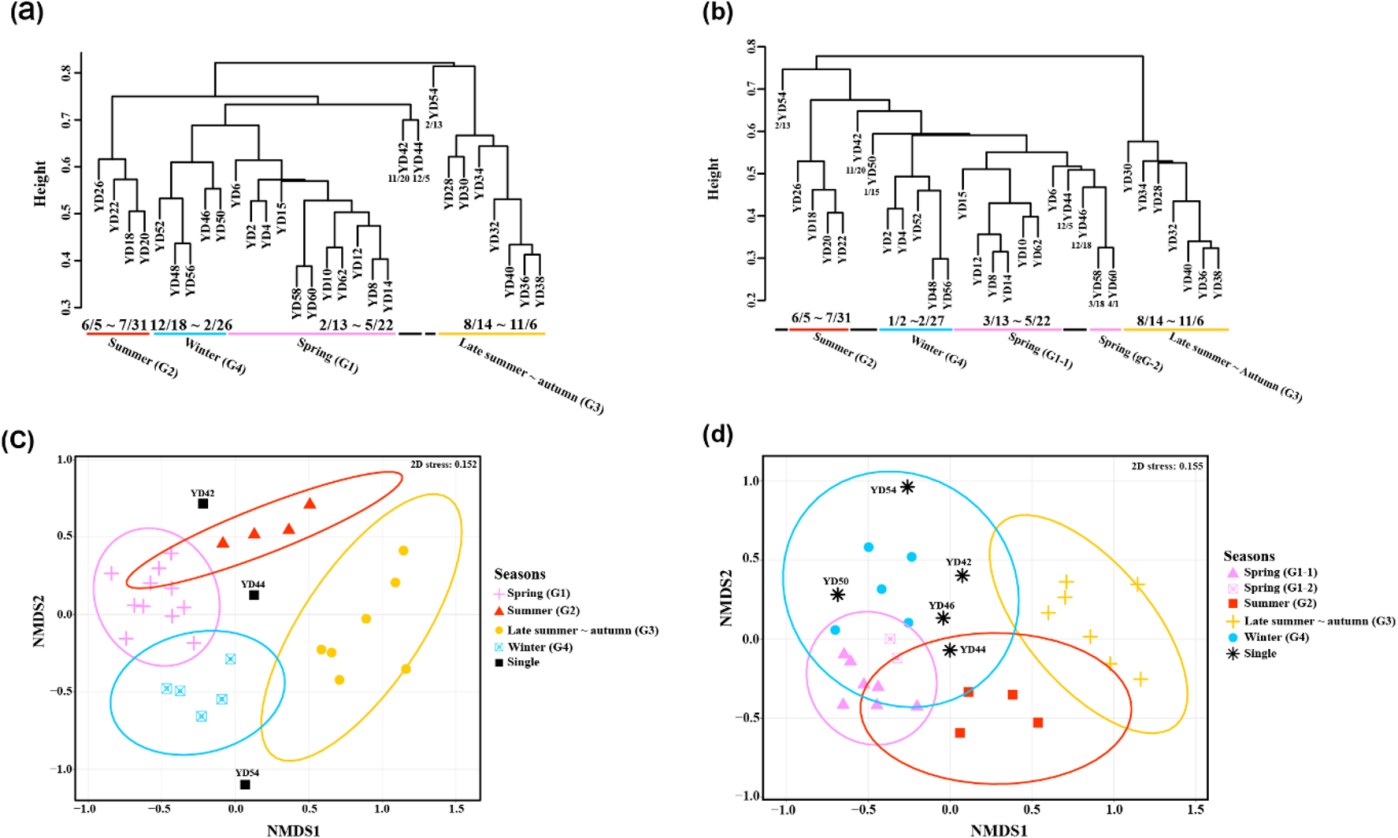
Results of the clustering analysis. Cluster dendrogram of (**a**) “total” zooplankton and (**b**) “common” zooplankton. NMDS plot of (**c**) “total” zooplankton and (**d**) “common” zooplankton. The colors of cluster dendrograms and the NMDS plot indicate the seasonally divided groups. Black indicates a single cluster with the minimum dissimilarity cutoff (“total” species: 0.68, “common” species: 0.56). Figures were produced using R (v4.0.3, https://www.R-project.org).

Clustering analysis was performed to investigate community structure changes over time (Fig. 6). Non-metric multidimensional scaling (NMDS) analysis [log_10_(*x* + 1) transformation, Bray–Curtis] of the “total” zooplankton was broadly divided into four main groups (dissimilarity cutoff 0.68) and three single-clustered samples (YD42, YD44, and YD54) (Fig. 6a, c). The four main groups were roughly divided temporally into the “spring” group (G1) (February 13 to May 22), “summer” group (G2) (June 5 to July 31), “late summer-autumn” group (G3) (August 14 to November 6), and “winter” group (G4) (December 18 to February 26) (ANOSIM significance = 0.001, *R* = 0.9221).

The same NMDS analysis was performed on the “common” zooplankton [log_10_(*x* + 1) transformation, Bray–Curtis] (Fig. 6c, d). Similar to the above, it was also largely divided into four main groups according to the season, but there were differences in some samples. The samples clustered in G2 and G3 were the same as those in the “total” zooplankton analysis. However, sample YD46 was included along with YD44 in G1 instead of G4, and G1 was divided into two subgroups (G1-1 and G1-2). Furthermore, there were three single-cluster samples (YD42, YD50, and YD54). Cluster analysis using only the “common” zooplankton did not well differentiate the temporal and seasonal differences in the zooplankton community compared with the “total” zooplankton analysis (ANOSIM significance = 0.001, *R* = 0.8785).

We then conducted a SIMPER analysis to determine each taxon’s average percentage contribution to each of the four seasonal groups [standardized log_10_(*x* + 1)-transformed data]. The top five highest contributing species to seasonal differences (*p*-value < 0.05) are indicated in Table 2. It was found that 15 species, except for the duplicates in the list, greatly contributed to the cluster structure variations according to time and season. Therefore, we confirmed their appearance to observe which seasonal group they represented. *P. leuckartii* (Branchiopoda), *E. nordmanni* (Branchiopoda), and *Eudactylopus yokjidoensis* (Copepoda) showed higher abundance in spring (G1) than in other seasons. *P. polyphemoides* (Branchiopoda), *B. trigonus* (Cirripedia), *Membranipora villosa* (Bryozoa), and *C. challengeri* (Cirripedia) were appeared in summer (G2) compared to other seasonal groups. A large number of *A. omorii* (Copepoda) represented throughout all seasons but their abundance decreased in late summer-autumn (G3). *Amphibalanus amphitrite* (Cirripedia), *B. intermedius* (Malacostraca), *Liriope tetraphylla* (Cnidaria), *S. doederleini* (Echinodermata), and *Paracalanus gracilis* (Copepoda) were the representatives of late summer-autumn (G3) species. Finally, *Clausocalanus furcatus* (Copepoda) appeared in greater abundance in winter than in other seasons (Fig. 5; Table 2). All top 5 taxa which contributed significantly to distinguishing each seasonal group were included in the “common” taxa. The result of the full SIMPER analysis is attached (Supplementary Analysis S1).

**Table 2:** The top five highest average contributing taxa to each of the four seasonal groups.

Taxa	Contribution (± SD)%	Average of G1	Average of G2
**“Spring” (G1) *vs*. “Summer” (G2)**			
Pleopis polyphemoides**	3.21 (0.95)	0.09	3.37
Balanus trigonus*	2.11 (1.23)	0.87	2.75
Eudactylopus yokjidoensis*	1.89 (1.37)	2.01	0.00
Podon leuckartii**	1.70 (0.94)	1.76	0.00
Membranipora villosa**	1.62 (0.99)	0.00	1.69

Taxa	Contribution (± SD)%	Average of G2	Average of G3
**“Summer” (G2) *vs*. “Late summer-autumn” (G3)**			
Pleopis polyphemoides*	2.05 (0.79)	3.37	0.46
Amphibalanus amphitrite*	1.98 (0.92)	0.76	3.60
Belzebub intermedius**	1.87 (0.67)	0.00	2.60
Liriope tetraphylla*	1.85 (0.94)	0.00	2.60
Schizaster doederleini*	1.67 (1.10)	0.30	2.53

Taxa	Contribution (± SD)%	Average of G3	Average of G4
**“Late summer-autumn” (G3) *vs*. “Winter” (G4)**			
Amphibalanus amphitrite**	2.94 (0.60)	3.6	0.00
Belzebub intermedius**	2.20 (0.84)	2.6	0.00
Acartia omorii**	2.13 (1.04)	1.15	3.64
Liriope tetraphylla**	2.07 (1.17)	2.6	0.14
Schizaster doederleini**	1.90 (1.31)	2.53	0.44

Taxa	Contribution (± SD)%	Average of G4	Average of G1
**“Winter” (G4) *vs*. “Spring” (G1)**			
Evadne nordmanni**	3.27 (1.60)	0.06	2.77
Clausocalanus furcatus**	2.58 (1.74)	2.16	0.23
Paracalanus gracilis*	2.53 (1.75)	2.35	0.50
Chthamalus challengeri*	2.52 (1.54)	1.15	2.75
Eudactylopus yokjidoensis*	2.35 (1.71)	0.00	2.01

Significant *p*-values (< 0.05 and < 0.01) are marked with asterisks (* and **).

### Searching for candidates of potential invasive species

In order to evaluate the reliability of the overall metabarcoding classification, the results of our analysis were compared with the national list of marine species (NLMS)^26^. Of the 267 species identified in our data (including sequence-read depth < 10), ~ 75.7% (202/267) was confirmed to be the correct taxonomic name (species level: 192; genus level: 9; family level: 1) by NLMS and ~ 24.3% (65/267) of the remaining species were not found (Table S8). One of them was a freshwater taxon (*Cyclops vicinus*)^27,28^ and 39 species in our data were only the same genus name in NLMS (Table S8). Moreover, 26 taxa did not have genus names as well as species names. *C. vicinus* is suggestive of debris that probably inflowed from the Nakdong River^29^. We confirmed whether the COI sequences for the 38 species that only existed in the NLMS with the same genus name had their congeneric species registered in NCBI. Twenty taxa of the 38 species have COI sequences for their congeneric species in the NCBI database. The remaining 18 taxa do not have COI sequences registered for any congeneric species in NCBI. Therefore, in the 18 cases, there might be a misannotation caused by a lack of sequence information. As a result, a final total of 46 taxa, 26 taxa without both species and genus name in NLMS and 20 taxa that have only the same genus name in NLMS and do not have the COI sequence for all other congeneric species within NLMS, was listed as candidates for potential invasive species or NIS (Table S8). It would be worth exploring the taxonomic identification in future studies.

## Discussion

In the study area (Yeongdo-gu, Busan), zooplankton diversity was highest in autumn (October) and lowest in spring (April) (Fig. 2; Table S4). This seasonal pattern was similar to previous observations of the zooplankton community in the Busan Bay and the southern coast of Korea^24,25^. In addition, the seasonal pattern is thought to have a relatively higher correlation with water temperature than salinity^30^. Copepod species dominated the zooplankton composition, followed by cirripedian larvae and branchiopods (Figs. 3 and S4). In previous studies, copepods were most dominant in the zooplankton communities in the coastal regions, followed by branchiopods or Cirripedia larvae, depending on the season or environment^24,25,31^.

It was confirmed that 30 temporal samples were roughly divided by season into four groups (Fig. 6). In addition, the 14 species that contributed significantly to each seasonal group as a result of SIMPER analysis were *Podon leuckartii* (Cladoceran), *Evadne nordmanni* (Cladoceran), *Eudactylopus yokjidoensis* (Harpacticoida), *Pleopis polyphemoides* (Cladoceran), *Balanus trigonus* (Sessilia), *Membranipora villosa* (colonial marine bryozoan), *Chthamalus challengeri* (Sessilia), *Acartia omorii* (Calanoida), *Amphibalanus amphitrite* (Sessilia), *Belzebub intermedius* (Decapoda), *Liriope tetraphylla* (Cnidaria), *Paraster doederleini* (Sea urchins), *Paracalanus gracilis* (Calanoida), and *Clausocalanus furcatus* (Calanoida). Note that species names are followed by WoRMS (http://www.marinespecies.org). On the southern coast of Korea, *P. leuckartii* is most abundant in April and reported to be negatively correlated with water temperature and salinity^31,32^. Likewise, in our study, *P. leuckartii* was most abundant in spring (G1) and not detected in summer (G2) when the water temperature was high (Fig. 5; Table S7). *E. nordmanni*, which is known to appear briefly in the spring when the water temperature is between 10 and 17 °C^31,33^, was analyzed as a representative of spring (G1; February to May) in our study (Fig. 5; Table S7). *E. yokjidoensis*, a new species reported in 2018, was collected from the southern coast of Korea in April 2016^34^. It showed high abundance in spring (G1), indicating that this new species may exist in our study area, but very little was found in other seasons in our samples.

*P. polyphemoides* (Cladoceran) appears throughout the year in Chinhae Bay, Korea although its abundance is especially high when the water temperature is 18 °C^33^. It was also reported in the Mediterranean Sea at 18–19 °C^35^. Similarly, it was found in summer (G2; June to July) within a temperature range of 17.4 to 21.8 °C in our study, representing this season. *M. villosa* (Colonial marine bryozoan) was reported in Busan during the summer (June) and was mainly distributed in coastal ports of Korea in summer and autumn (August to November)^36^. In our data, it appeared only in summer (G2).

*L. tetraphylla* and *B. intermedius*, representatives of late summer-autumn (G3; August to November) in our study, have not already been accurately modeled for their annual distribution on the southern coast of Korea. *L*. *tetraphylla* was only detected in the coastal region of Busan in late September^37^ and *B. intermedius* was confirmed only in the southern Yellow Sea of Korea during October^38^.

*P. gracilis* and *C. furcatus* were dominant in late summer-autumn (G3) and in the winter group (G4), supporting previous studies^39–41^. *A. omorii* was dominant throughout all seasons but with relatively low abundance in G3. *A. omorii* is not reported to appear in the summer when the water temperature is high (average 24 °C)^24^. The high-water temperatures in August may explain this species’ disappearance in September and its low abundance in late summer-autumn (G3) (Figs. 5 and S2). In addition, this species frequently appears in the eutrophic inner bay^24,42^. Therefore, it indirectly shows that our study area, the entrance to Busan Port, may have undergone some degree of eutrophication.

Cirripedia larvae *B. trigonus* and *C. challengeri* were most abundant in summer (G2) and *A. amphitrite* in late summer-autumn (G3), respectively. It has been reported that *C*. *challengeri* appeared most intensively in August–October near Oryuk Islets off Busan^43^, the outer area of our study area. However, because it is difficult to classify Cirripedia larvae down to the species level, no seasonal changes in the distribution of the other two Cirripedia species have been reported. According to a previous study, Cirripedia larvae are relatively abundant in summer and autumn than in other seasons^31^. Finally, *S. doederleini* is mainly distributed in the Caribbean^28,44^, and no record was found in Korea.

Our metagenomic analysis results revealed that the seasonal zooplankton community could be largely divided into four groups corresponding to the four seasons. The distribution pattern of species representing each seasonal group has shown to be largely consistent with past research^24,25^. It was also possible to estimate Cirripedia larvae species, which was not identified in previous studies. Given these results, the study of marine zooplankton community and diversity by metabarcoding is efficient and enhances understanding of the dynamics of the zooplankton community throughout the year. Moreover, should the metabarcoding sequence data and the analyzed results be stored and remain available for future analyses, it will allow easy detection of changes in species composition and any introduction of invasive species into the Busan Port ecosystem by simply uploading their COI sequences to the database.

Most of the species not recorded in NLMS (average read counts per sample: 23.74) showed relatively lower abundance than the identified species (119.85). For this reason, they may have been relatively rare in the coastal region of Korea and difficult to find. In addition, these species may pose a potential threat to marine ecosystems as invasive species, introduced by ship movements or climate change^45^. Therefore, in future monitoring of zooplankton in the region, it is necessary to investigate these species’ presence or absence carefully. Adding the presence or absence of barcode sequences (e.g., COI, 18S rRNA, ITIS) and database registration information to the NLMS in the future can greatly contribute to the improvement of the accuracy of future studies using metabarcoding. With only one year of observation, although it is difficult to state these are early invasive species in the Busan Port ecosystem, if we monitor them for a long time using metabarcoding, it should reveal their appearance trends. Hence, it may be possible to judge whether their abundance is increasing or just a short-term influx.

All 64 taxa identified in the “common” zooplankton were included in the “total” zooplankton taxa and accounted for 24.0% (64/267) of “total” zooplankton species. The small number of taxa in the “common” zooplankton accounted for about 97.8% (2,485,517/2,542,332) of the final read count for “total” zooplankton. Some zooplankton taxa occupy most of the abundance in the study area, and a large number of the other taxa show a very low frequency of appearance. Even if only “common” zooplankton was used, similar to the use of “total” zooplankton, the change in the temporal community structure was divided into the four seasonal groups. Moreover, in the SIMPER analysis, the top 5 species that showed significant differences among the seasons were included in the “common” zooplankton (Fig. 6; Table 2). Nonetheless, if we include the species that occupied a small proportion in the analysis, it provides a better distinction of seasonal changes in community composition (Fig. 6). The inclusion of rare species also helps detect early invaders or NIS introduced by climate change or human activities to predict and prepare for their impact on the ecosystem. Therefore, a species with low abundance should be reflected in the ecological analysis after sufficient data curation.

Continuous and extensive zooplankton ecological monitoring studies involving metabarcoding methods have several advantages. First, this method can be more efficient than traditional methods^46^. As exemplified by *E*. *yokjidoensis* in our study, if the researchers only register the COI (or any other marker sequence) for a new species that has just been reported, it is possible to quickly screen and predict where the new species is distributed within the stored metagenomic library data. Although morphological methods can produce similar results by re-analyzing previously-stored samples, this work requires relatively more experts’ labor than the metagenomic methods. Second, it can be possible to classify zooplankton larvae difficult to identify morphologically, like Cirripedia larva. Lastly, species that are difficult to detect by any morphology-based methods due to very small populations, such as early invaders and NIS candidates, can be detected with high sensitivity by metagenomics^47^. Nonetheless, even with metagenomic methods, it is hard to distinguish whether a species is a real member of the study area or a fragment of a dead specimen flowed from a river such as *C. vicinus*, a freshwater species identified in our data. Therefore, complementing metagenomic analysis with traditional morphology will enable understanding marine ecosystems more specifically and clearly than either approach alone, especially in extensive and continuous ecosystem monitoring.

## Conclusions

Our study investigated how to maximize the advantages of metabarcoding for monitoring of zooplankton community structure and diversity in urban coastal regions like the entrance of Busan Port, Korea. In this study, the zooplankton community showed a typical four-season pattern and the species representing each seasonal group were generally consistent with previous studies. Even after the rare species were removed, “common” zooplankton enabled us to confirm the approximate pattern of change in zooplankton diversity. However, using all the OTUs, “total” zooplankton yielded a relatively more pronounced seasonal change in the zooplankton community structure, and potential candidates for early invasive species in the port ecosystem were identified. Although our observations were conducted over a relatively short period at one sampling station, it suggests that regular monitoring of urban coastal areas by metabarcoding could be useful for understanding this ecosystem and detecting potential hazards. Furthermore, it is expected that the accumulation of monitoring data using metabarcoding will enable predicting and responding quickly to changes in zooplankton diversity.

## Material and methods

### Sampling sites and collection

The samples were collected at about 2-week intervals from February 13, 2019, to April 16, 2020, from a sampling site in Busan Bay (35.077° N, 129.083° E) off the southern coast of Korea, near the Korea Institute of Ocean Science & Technology (KIOST) (Fig. 1; Table 1). A plankton net with 200-μm mesh and 60-cm opening diameter was towed horizontally for a distance of 100 m (total filter volume, 20.6 m^3^) across the water surface (< 1 m depth) for zooplankton sampling. Temperature and salinity were measured at 0.5 m and 1.0 m depth using a conductivity meter (YSI 30, OH, USA) and the average of these two values was used for further analysis.

### DNA isolation and amplification by PCR

The collected zooplankton sample was transferred to the laboratory, where DNA extraction was undertaken immediately from 10 mL of the approximate 200-mL sample. The rest of the sample was stored in alcohol for later use. The TIANamp Marine Animals DNA Kit (Tiangen Biotech, China) was used to isolate DNA from the zooplankton sample.

The gene for eukaryotic mitochondria cytochrome *c* oxidase I (COI) was amplified by using the degenerate primer set mlCOIintF (5′-GGWACWGGWTGAACWGTWTAYCCYCC-3′) and jgHCO2198 (5′-TAIACYTCIGGRTGICCRAARAAYCA-3′)^48^. Amplification reactions were performed in 0.2-mL PCR tubes in a 30-μL mixture containing 1.8 μL of 1 × 10^–5^ μM of primers, 2 μL of DNA template, 11.8 μL of ultrapure water, 15 μL of 2X DNA-free Taq Master Mix (CellSafe, Korea). Samples were amplified for 40 cycles using a MaxyGene Gradient Thermal Cycler (Axygen, CA, USA) under the following conditions: initial denaturation at 95 °C for 5 min (1 cycle), denaturation at 95 °C for 30 s, annealing at 46 °C for 30 s, and extension at 72 ℃ for 60 s. The final extension was performed at 72 ℃ for 5 min. A negative control (without DNA template) was performed for the PCR step to detect potential contamination. The PCR product was purified using the Universal DNA Purification Kit (Tiangen Biotech, China). Quantity and quality analyses of the PCR amplified fragments and purified product were estimated using capillary electrophoresis and an ND-1000 spectrophotometer (Nanodrop, Power Lab, Korea). To ensure a homogeneous number of sequencing reads from each sample, 100 ng of each amplicon DNA was taken and diluted to 4 nM with determination of the size of the DNA with Agilent Technologies 2100 Bioanalyzer (DNA 1000 Chip, USA). All the diluted samples were pooled and used in end-repair and ligation of adaptors followed by sequencing in the MiSeq platform according to the manufacturer’s protocol. Next-Generation Sequencing library constructed using the Nextera XT Index Kit and the TruSeq Nano DNA Sample Prep Kit as the main capture kit and sequenced using the MiSeq platform were performed at Theragen facilities (Theragen Biotech, Korea).

### Quality control and merge

To filter low-quality reads, cutadapt (ver. 2.8)^49^ was used to remove amplicon sequences and to discard any unknown nucleotide “N” and reads that had no primer sequences or < 200 bp in length. To maximize the read depth for each sample, low-quality score cutoff values were set differentially in forward-end reads (*q* = 30) and reverse-end reads (*q* = 20). Reads without a mate (singletons) were discarded using the pairfq script (ver. 0.17.0; https://github.com/sestaton/Pairfq). Merging of paired-end reads was conducted by pear (Paired-End reAd mergeR, ver. 0.9.2)^50^ with the following parameters^51^: *v* = 30, *t* = 50, *n* = 250, *m* = 350, and *q* = 20.

### OTU clustering

The resulting FASTA files were clustered using the vsearch tool (ver. 2.7.0)^52^. Next, sequences were dereplicated, sorted (-derep_fulllength), and those with < 2 clusters (singleton) were removed. The outputs that passed the previous steps were pre-clustered at a similarity threshold of 99% (-cluster_size). After pre-clustering, chimeras were de novo detected and removed using the UCHIME algorithm (-uchime_denovo)^53^. Lastly, final OTUs were clustered at a similarity threshold of 98% (-cluster_size) from pre-clustered OTUs, and all sequences were assigned to OTUs. A flowchart of the steps involved in metagenomics analysis is given in Fig. S1.

### Taxonomic identification

Taxonomy was assigned to the OTU table using blastn of the Basic Local Alignment Search Tool (BLAST, ver. 2.10.0 +)^54^ against the National Center for Biotechnology Information (NCBI, http://www.ncbi.nlm.nih.gov) non-redundant nucleotide (NCBI nr/nt) database (as of August 26, 2020, N = 60,251,963 sequences) with an E-value < 1 × 10^–10^, database size of 3 × 10^11^, and percentage identity ≥ 99% options for genus or species level. The following three steps were performed on the BLAST results to increase the accuracy of taxonomic assessment and eliminate chimeric OTUs: (1) accept only OTUs having a length of gaps and mismatches ≤ 5; (2) accept only aligned lengths ≧200 bp and bit-score ≧100; (3) select one with the longest alignment length if there are many OTUs aligned with the same query sequence. The remaining OTUs were then processed for final taxonomic identification.

The taxonomic assignment and hierarchical classifications from NCBI accession numbers were done using the ‘taxonomizr’ package^55^ in R software. Next, the OTU tables were modified to the species or genus level (in case that the NCBI database did not contain species level information) and were used for further analyses. After BLAST, all OTUs corresponding to the shown taxa were identified using a custom Perl script (eDNA_shell_fast_taxonomy.pl). The Perl script is available upon request from the authors. In addition, ‘Bacteria’, ‘Fungi’, ‘Fish’, ‘Insect’ (from inland), ‘Mammalian’, ‘Phytoplankton’, and ‘Environmental’ or ‘Unclassified’ OTUs were removed from the OTU count table because we focused on marine zooplankton diversity. Finally, the synonymized taxa were combined into one species by referring to the World Register of Marine Species (WoRMS, http://www.marinespecies.org)^28^.

### Biodiversity analysis

In zooplankton studies using metagenomics, it is common that low-abundance reads are discarded, and further analysis is performed to remove contamination or PCR artifacts or compare morphological analysis data with the metagenomically dominant species^3,56,57^. Instead, in this study, we applied two methods to determine the difference between the data with the low-abundance OTUs removed (“common” zooplankton) and the data with all OTUs (“total” zooplankton). To confirm the difference between the two methods, the OTU removal standard used in the high contamination risk sampling method was followed. Thus, for “common” zooplankton, OTUs that contributed > 0.5% of sequences in at least one sample were retained^3^. Conversely, “total” zooplankton was used for analysis without removal of low-coverage OTUs.

All samples were rarefied at the lowest sequencing depth to reduce biases resulting from differences in sequencing depth using vegan^58^ in R software. As there were many un-assigned OTUs in taxonomic assessment, rarefaction was performed at the OTU level to secure as much read depth as possible for “total” zooplankton. For “common” zooplankton, rarefaction was performed at the remaining species level after taxonomic assessment. Using phyloseq^59^ in R software, species richness (observed or Chao1 index) and Shannon diversity index were estimated. Linear regression models were used to examine the relationship between environmental variables and biodiversity with R software^60^.

NMDS was employed to cluster samples according to seasonally different community compositions using the phyloseq and vegan packages in R. The original species-level OTU data were transformed to log_10_(abundance + 1) before NMDS. Then, NMDS for transformed data was conducted using the Bray–Curtis distance method (100 permutations). Analysis of similarity (ANOSIM) was applied to test each seasonal group’s significant effects on community composition (999 permutations). Similarity percentages (SIMPER) analysis was conducted on Bray–Curtis similarities from log_10_(abundance + 1) transformed (100 permutations) data using the vegan package implemented in the R programming language^58,60^. All figures (except for Figs. 1, S1, and S2) were initially created using R^60^.

By comparing the species shown in our analysis results with NLMS published by the National Marine Biodiversity Institute of Korea^26^, we confirmed the species listed as candidates for NIS. First, all the species identified by metabarcoding were checked if any records had appeared in the coastal region of Korea. For the species in our results that did not exist in NLMS, the congeneric species in NLMS were checked if they had taxonomy information and COI sequence registered in the NCBI. Finally, the taxa without both species and genus name in NLMS plus COI sequence for all other congeneric species within NLMS were estimated as potential early invader species or NIS.

## Supplementary Information

Supplementary Figures.

Supplementary Tables.

Supplementary Information.

## Data Availability

All sequencing data are archived in the NCBI Sequence Read Archive (SRA) database under BioProject number PRJNA739266.
