# Supplementary material for: Zooplankton diversity monitoring strategy for the urban coastal region using metabarcoding analysis
Source: Sci Rep. 2021 Dec 21;11:24339. doi: 10.1038/s41598-021-03656-3 (PMC8692418; doi:10.1038/s41598-021-03656-3)
Supplement: Supplementary file 1 — Supplementary Figures. [file 41598_2021_3656_MOESM1_ESM.docx]

**SUPPLEMENTARY FIGURES**

**Zooplankton diversity monitoring strategy for the urban coastal region using metabarcoding analysis**

Chi-une Song^1^, Hyeongwoo Choi^1^, Min-Seung Jeon^1^, Eun-Jeong Kim^1^, Sung Kim^2^, Choong-gon Kim^2^, Hyenjung Hwang^2^, Dayu Wiyati Purnaningtyas^2,3^, Seok Lee, Seong-il Eyun^1*^, Youn-Ho Lee^2*^

^1^Department of Life Science, Chung-Ang University, Seoul 06974, Korea

^2^Korea Institute of Ocean Science and Technology, Busan 49111, Korea

^3^KIOST School, University of Science and Technology, Busan 49111, Korea

**
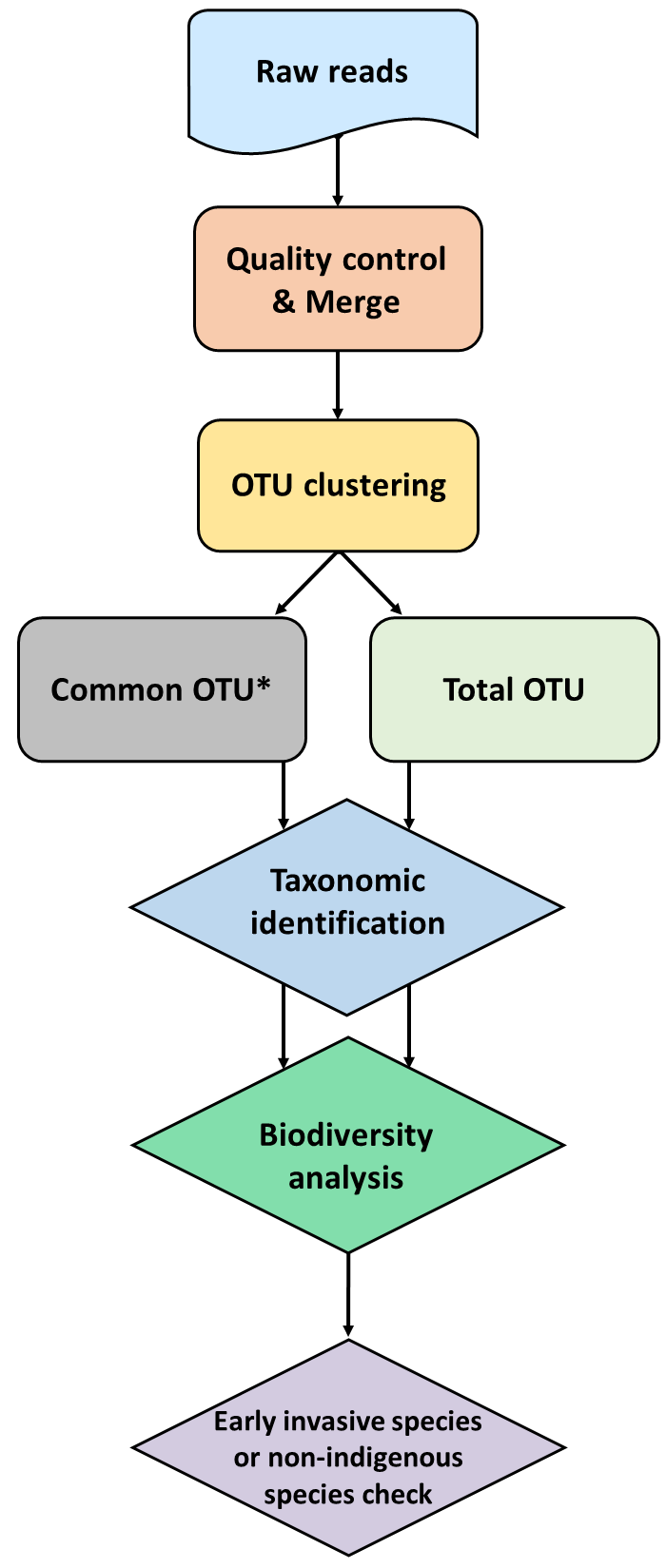
**

**Figure S1. Schematic Flowchart of the metagenomics analysis in this study.**

**
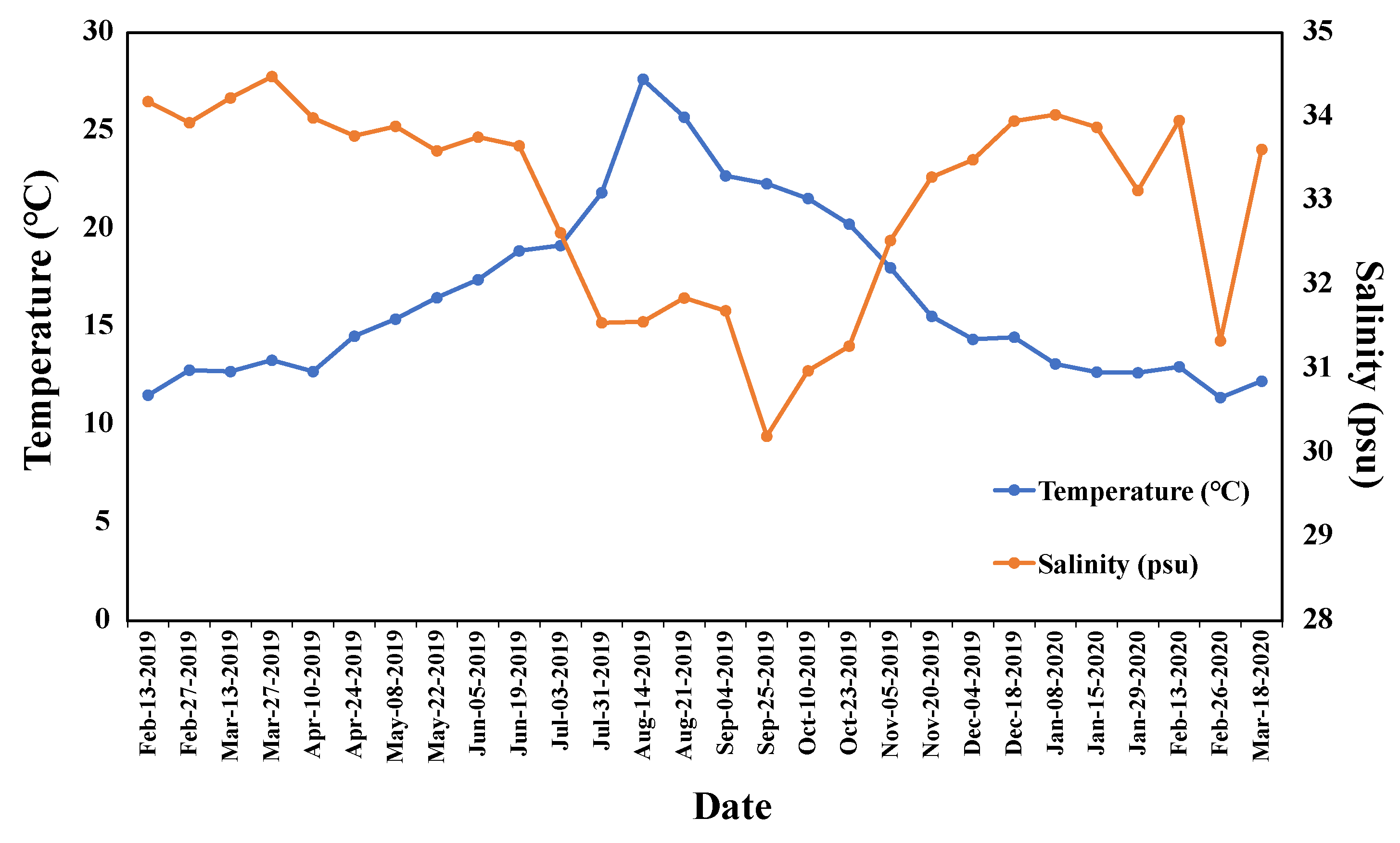
**

**Figure S2.** **Temporal distribution of the environmental conditions.** The blue marks and lines indicate temperature (℃) and orange indicates salinity (psu).


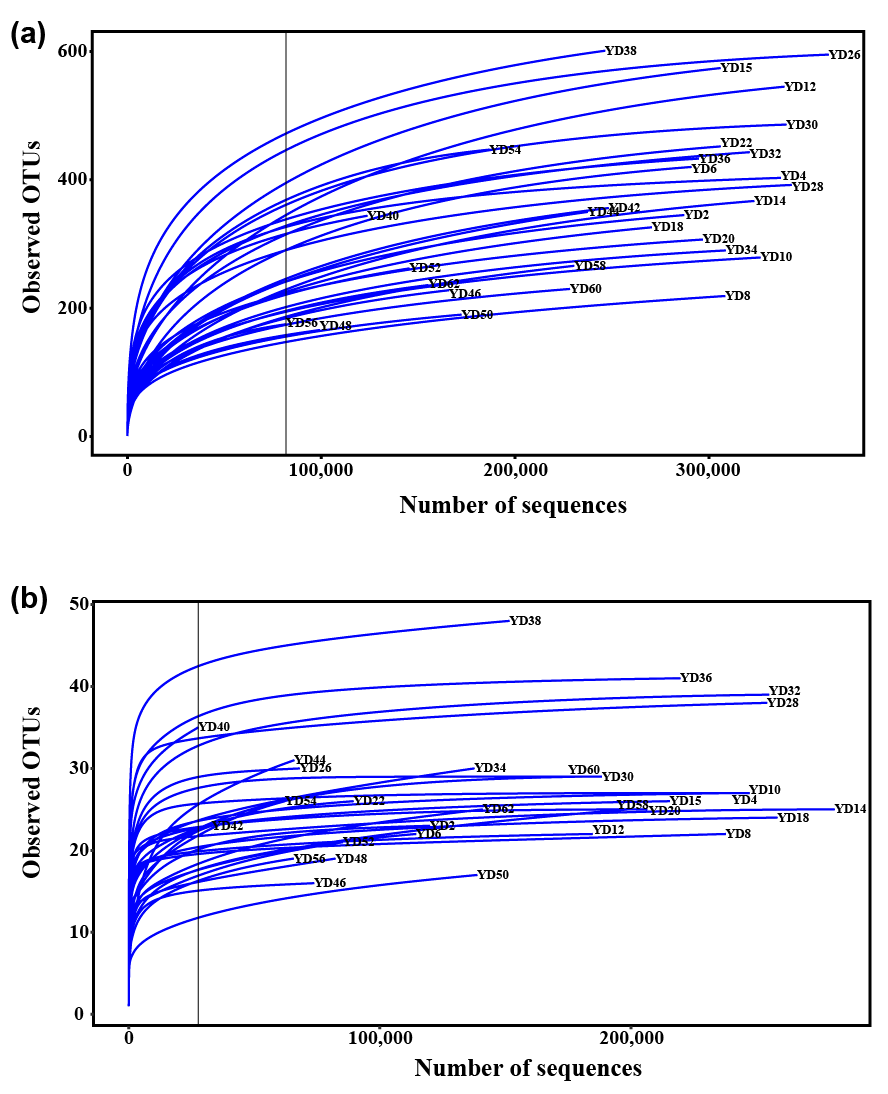


**Figure S3.** **Rarefaction curve for “common zooplankton” and “total zooplankton” samples.** (**a**) Rarefaction curve for “total zooplankton” sample with OTU level. (**b**) “Common zooplankton” samples for zooplankton level. Black vertical lines indicate the minimum sample size. Figures were produced using R (v4.0.3, https://www.R-project.org).


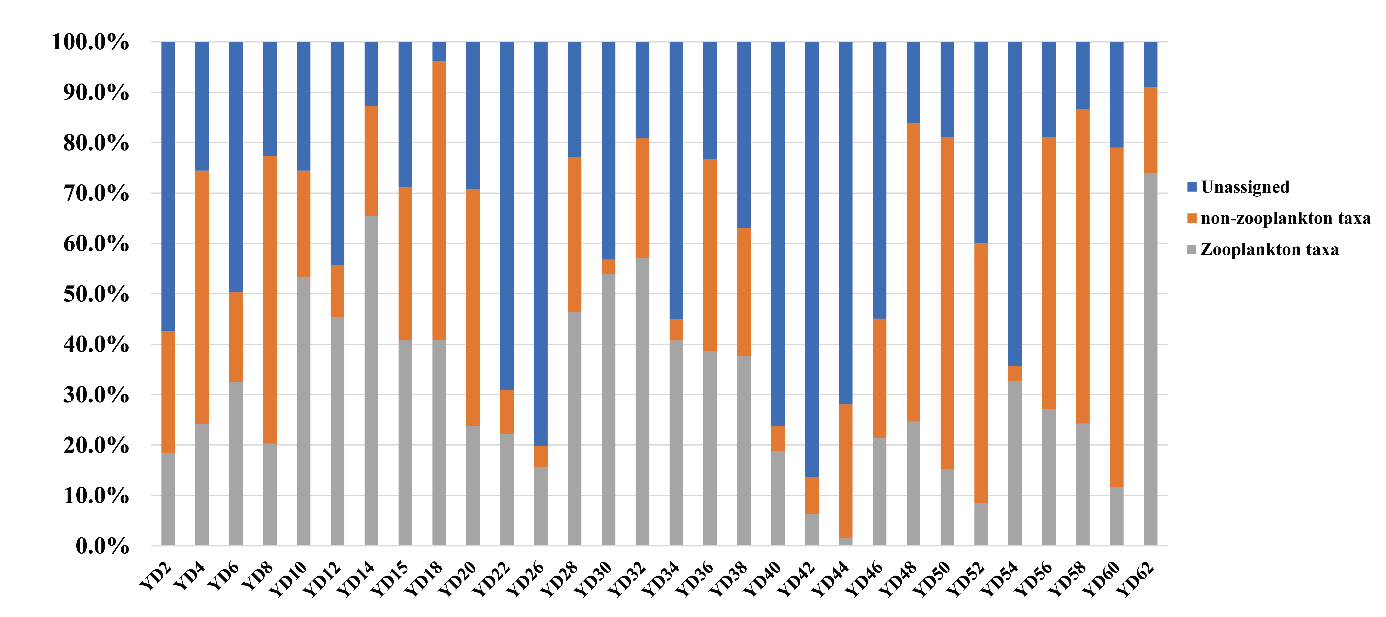


**Figure S4.** **Proportion of reads for the zooplankton communities**. Figures were produced using R (v4.0.3, https://www.R-project.org).


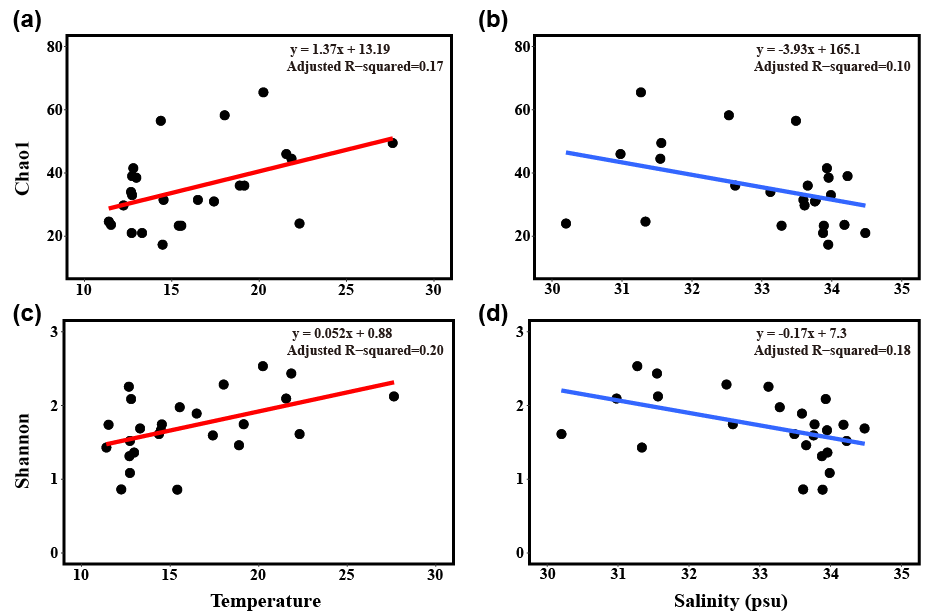


**Figure S5.** **Linear correlation between the α-diversity index and environmental conditions.** (**a**) Chao1 index and temperature. (**b**) Chao1 index and salinity. (**c**) Shannon index and temperature. (**d**) Shannon index and salinity. Regression equations and the adjusted R^2^ values are shown in the plot. Figures were produced using R (v4.0.3, https://www.R-project.org).


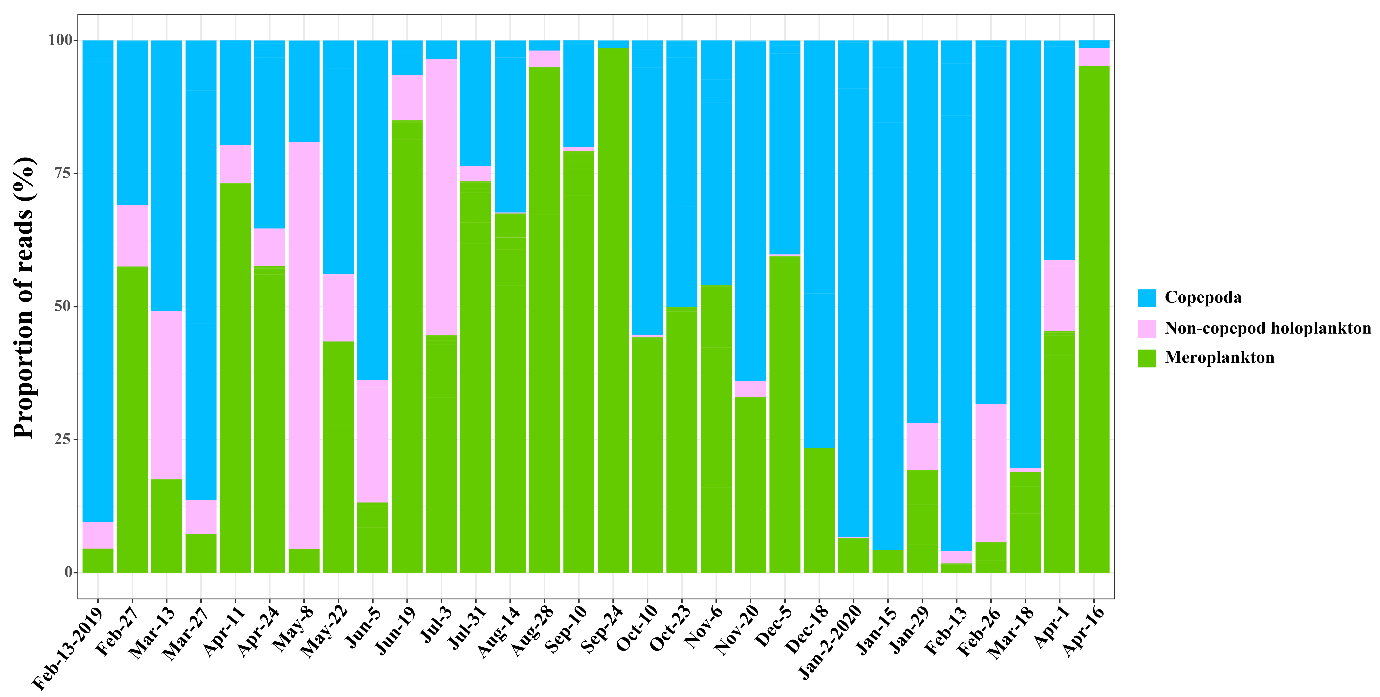


**Figure S6. Seasonal pattern of the three main groups (copepods, meroplankton, and non-copepod holoplankton).** Figures were produced using R (v4.0.3, https://www.R-project.org).


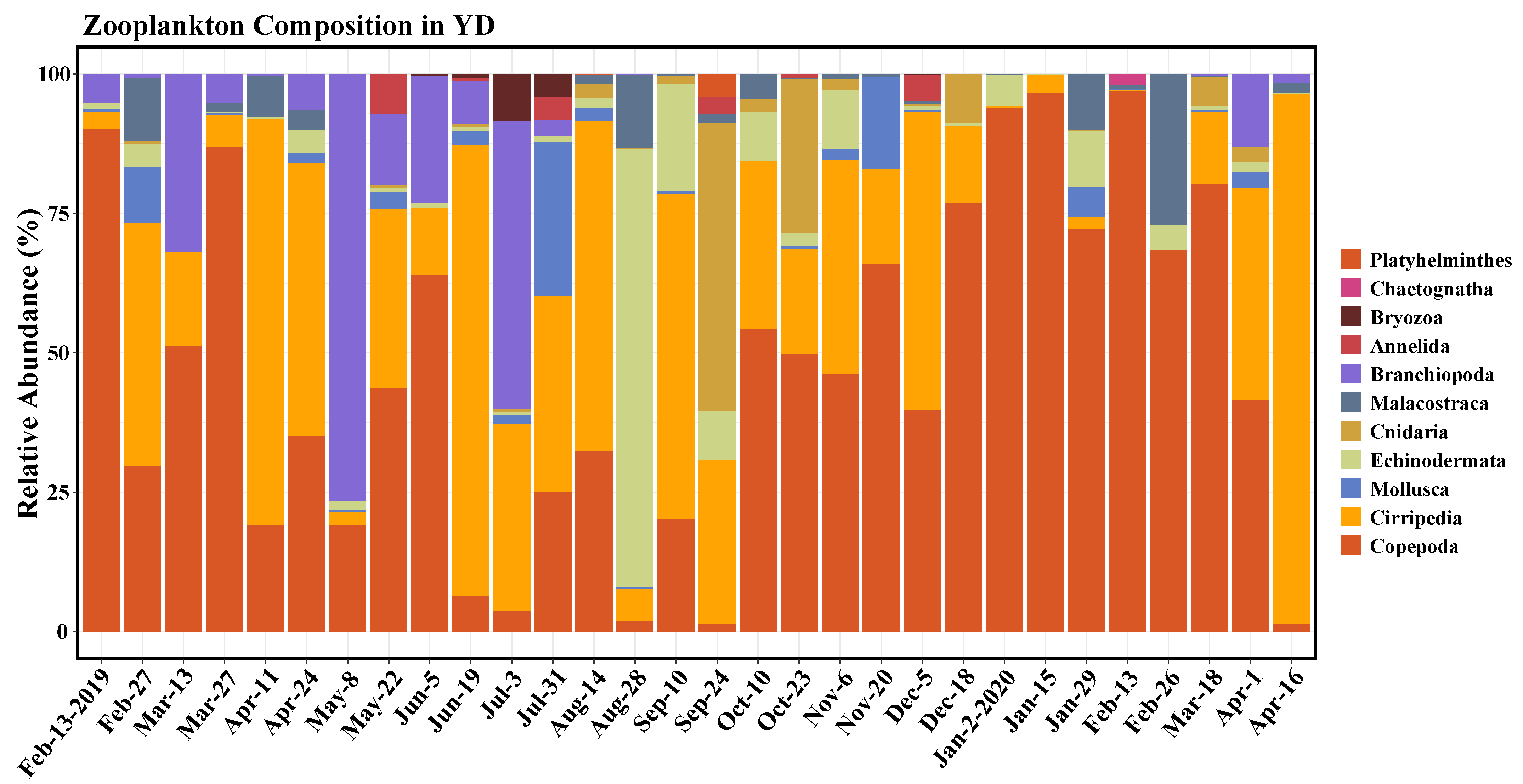


**Figure S7.** **Taxonomic composition of zooplankton for 30 “common zooplankton” samples with 11 taxonomic groups.** The relative abundances were calculated as the proportion of corresponding group’s read counts to the total number of reads in the samples. Figures were produced using R (v4.0.3, https://www.R-project.org).


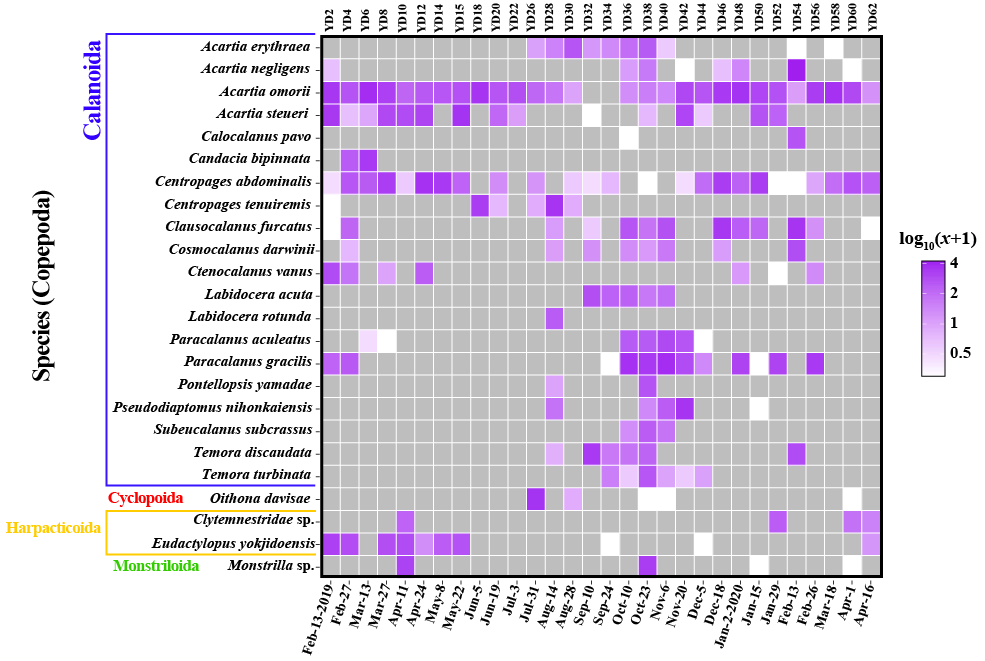


**Figure S8.** **Heatmap of subclass Copepoda for common species.** Each read count is transformed log_10_(abundance+1). The colors indicate relative abundance from high (purple) to low (white) and gray is 0. Figures were produced using R (v4.0.3, https://www.R-project.org).
